# Supplementary material for: Assembly of minimally fragmented metagenomic pangenome graphs
Source: bioRxiv. 2026 Oct 30:2026.10.29.762972. Preprint. [Version 1] doi: 10.64898/2026.10.29.762972 (PMC13734104; doi:10.64898/2026.10.29.762972)

bioRxiv preprint doi: https://doi.org/10.64898/2026.10.29.762972; this version posted October 30, 2026. The copyright holder for this preprint (which was not certified by peer review) is the author/funder, who has granted bioRxiv a license to display the preprint in perpetuity. It is made available under a CC-BY 4.0 International license.

Supplementary Figures

Supplementary Figure S1. Derivation of the genus-level TANI threshold. **(A)** Distribution of TANI edge weights across MST edges. **(B)** Percolation plateau over the representative TANI matrix, with threshold of 0.397 highlighted. **(C)** Distribution of pairwise TANI estimates across all intra-subtree pairs and pairs constrained to MST edges.

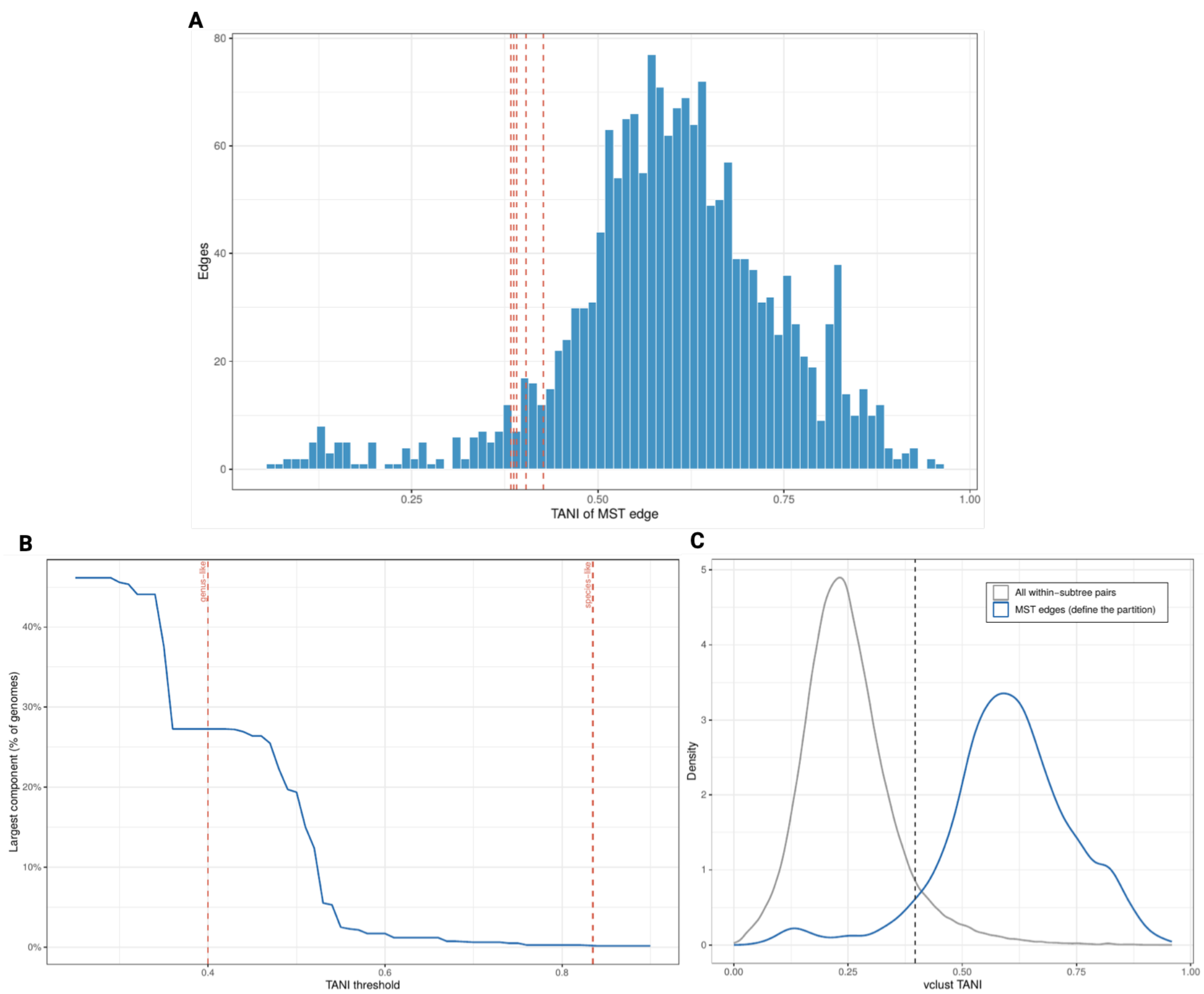

bioRxiv preprint doi: https://doi.org/10.64898/2026.10.29.762972; this version posted October 30, 2026. The copyright holder for this preprint (which was not certified by peer review) is the author/funder, who has granted bioRxiv a license to display the preprint in perpetuity. It is made available under a CC-BY 4.0 International license.

Supplementary Figure S2. Sensitivity of the assembly to genome ordering. Progressive max TANI vs. already embedded genomes observed during assembly of the representative genomes (N=1,749). **(A)** Five component-seeded orderings compared against five random orderings, alongside the isolated and length-sorted default VG msga orderings for the full set of core/representative genomes. **(B)** Max TANI vs. already embedded genomes over the initial set of genomes (N=349, 20%). In the left panel, the positional max TANI is shown while the right panel displays the distribution. In both panels, the mean max TANI for each seed is also shown.

A

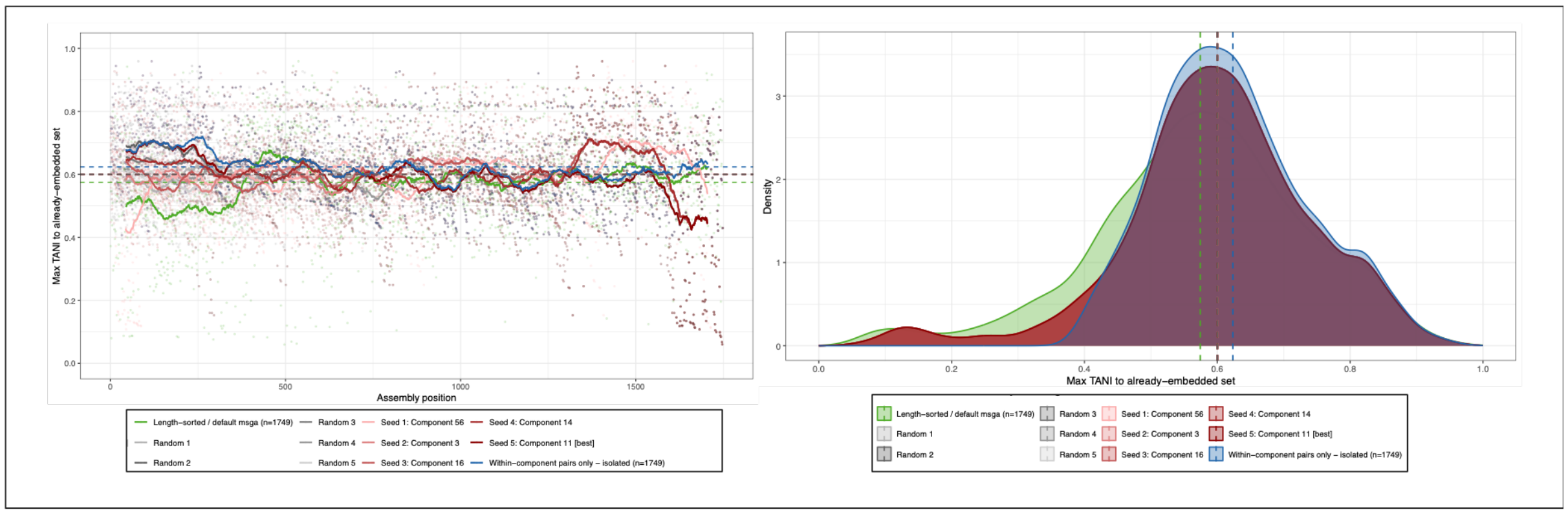

B

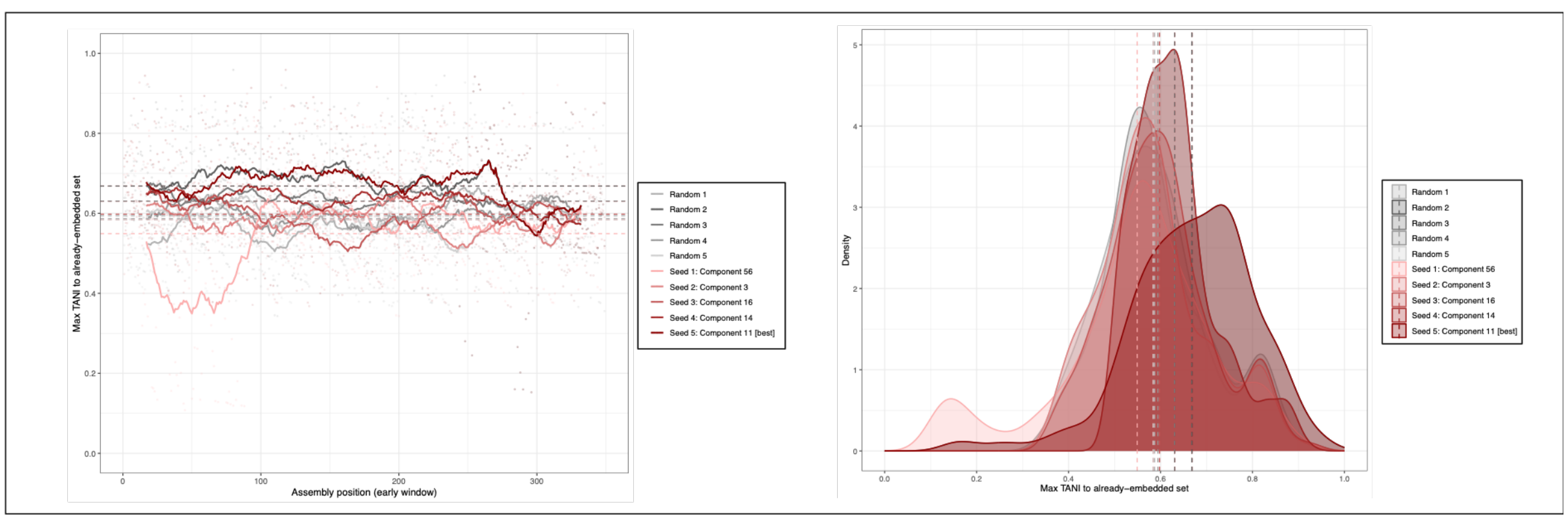

Supplement: Supplementary file 1 [file NIHPP2026.10.29.762972v1-supplement-1.pdf]
